# Transcranial Doppler findings in patients with COVID-19: a prospective observational study

**DOI:** 10.1055/s-0046-1825526

**Published:** 2026-07-14

**Authors:** Marcella Barreto Santos, Ana Flávia Silveira, João Brainer Clares de Andrade, Eva Carolina Rocha, Fabiano Moulin de Moraes, Hanna Nery Ferraz Martins, Daniela Laranja Gomes Rodrigues, Gisele Sampaio Silva

**Affiliations:** 1 Universidade Federal de São Paulo, Escola Paulista de Medicina, Departamento de Neurologia e Neurocirurgia, São Paulo SP, Brazil.; 2 Universidade Federal de São Carlos, Centro de Ciências Biológicas e da Saúde, Departamento de Fisioterapia, São Carlos SP, Brazil.; 3 Centro Universitário São Camilo, São Paulo SP, Brazil.; 4 Hospital Alemão Oswaldo Cruz, Responsabilidade Social, São Paulo, Brazil.; 5 Hospital Israelita Albert Einstein, Departamento de Neurologia, São Paulo SP, Brazil.

**Keywords:** Intracranial Embolism, Ultrasonography, Doppler, Transcranial, COVID-19, Monitoring

## Abstract

**Background:**

Transcranial Doppler (TCD) is a diagnostic tool to detect real-time spontaneous microembolic signals (MESs) in intracranial circulation. Critically-ill coronavirus disease 2019 (COVID-19) patients may present an increased likelihood of MESs due to systemic inflammation and hypercoagulability.

**Objective:**

To assess the presence of MESs and other TCD parameters, including cerebral blood flow velocity (CBFV), pulsatility, and resistance indices, and to calculate the estimated cerebral perfusion pressure (eCPP) in patients with and without COVID-19.

**Methods:**

The TCD monitoring was performed for 1 hour in both middle cerebral arteries (MCAs) of adult patients in the Intensive Care Unit (ICU) with confirmed COVID-19 infection. The eCPP was calculated by multiplying the mean arterial pressure (MAP) by the ratio of end-diastolic velocity to mean CBFV, then adding 14 mmHg.

**Results:**

We evaluated 31 subjects: 11 intubated COVID-19 patients, 10 non-intubated COVID-19 patients, and 10 intubated non-COVID-19 patients. Patient ages ranged from 29 to 84 (median: 62; interquartile range [IQR]: 50–70) years, with 64.5% being male individuals. A total of 17 (54.8%) subjects died during hospitalization, and 2 patients (6.45%) lacked an ultrasonographic signal via the transtemporal window. No MESs were detected. A positive correlation was found between the resistance index of MCAs ( *p* = 0.390) and D-dimer ( *p* = 0.049). In contrast, direct bilirubin ( *p* = -0.494), C-reactive protein (CRP; *p* = -0.540), activated partial thromboplastin time (aPTT; *p* = -0.495), and D-dimer ( *p* = -0.439) were negatively correlated with eCPP.

**Conclusion:**

No spontaneous cerebral microemboli were observed in this cohort, but patients with higher direct bilirubin, CRP, aPTT, and D-dimer presented lower eCPP.

## INTRODUCTION

Coronaviruses comprise a large family of viruses associated with a wide range of respiratory, enteric, hepatic, and neurological pathologies. 1 2 Severe acute respiratory syndrome coronavirus 2 (SARS-CoV-2), a member of the family *Coronaviridae* , was first identified in December 2019 in Wuhan, China. 3 On March 11, 2020, the World Health Organization (WHO) declared a pandemic in response to the rapid global increase in cases. 4

Numerous scientific publications have emerged during the pandemic, highlighting the neurological manifestations associated with SARS-CoV-2 infection and its variants. The mechanisms underlying these manifestations appear to be multifactorial, ranging from the viral tropism for angiotensin-converting enzyme 2 (ACE-2), 5 which is widely expressed in pulmonary parenchyma as well as in other tissues, including that of the nervous system, to immune-mediated processes and molecular changes secondary to systemic inflammatory responses. 6 7

In critically-ill coronavirus disease 2019 (COVID-19) patients, systemic inflammation, endothelial damage, and hypercoagulability may facilitate the generation of spontaneous microembolic signals (MESs) within the intracranial circulation. 8 In this context, transcranial Doppler (TCD), a diagnostic modality initially described by Aaslid in 1982, 9 is an invaluable tool to diagnose and monitor cerebrovascular diseases, as it enables a non-invasive evaluation of cerebral hemodynamics. 10 11 A practical examination, TCD enables the bedside monitoring of patients without requiring transportation to other areas, a particularly relevant advantage for COVID-19 patients, who are often clinically- and hemodynamically-unstable and in isolation. 12

The present study aimed to assess the frequency of spontaneous cerebral microembolism through non-invasive monitoring using TCD, as well as other TCD parameters, such as cerebral blood flow velocity (CBFV) in the middle cerebral arteries (MCAs), pulsatility index, resistance of the MCAs, estimated cerebral perfusion pressure (eCPP), and presumed non-invasive intracranial pressure (niICP). These parameters were correlated with COVID-19 severity markers 8 in a cohort divided into three groups of interest: patients with confirmed COVID-19 on mechanical ventilation (MV), patients with COVID-19 not requiring MV, and patients on MV for other diagnoses (COVID-19-negative).

## METHODS

### Study design and setting

We conducted a prospective, observational, single-center study in the inpatient units, including seven intensive care units (ICUs), at Hospital São Paulo (Universidade Federal de São Paulo, UNIFESP), a large tertiary teaching hospital in Brazil, from July 2020 to August 2021.

All data were collected during a single assessment using TCD, which was performed by the same experienced examiner, alongside data retrieved from the hospital's electronic medical records. Our convenience sample consisted of adults with confirmed COVID-19 infection, by reverse transcription-polymerase chain reaction (RT-PCR), requiring or not requiring MV. The onset of symptoms was within 14 days before study inclusion, and for patients on MV, the time from hospital admission to study inclusion was 72 hours. The non-COVID-19 MV group included patients on MV for non-neurological diagnoses, including respiratory failure and sepsis. The exclusion criteria were pregnant women, patients unable to undergo TCD monitoring (due to skin lesions and/or infections in the area of the probe) and/or simultaneous helmet monitoring application, individuals with a head circumference smaller than 47 cm, those who had previously undergone decompressive craniectomy, and patients with acute central or peripheral nervous system impairment during the evaluation period.

### Data collection

Data from the electronic medical records included clinical and epidemiological characteristics of the participants, such as age, sex, presence of risk factors, and comorbidities that may alter general TCD parameters and influence COVID-19 severity, as described in Table 1 . This data was used to calculate the Sequential Organ Failure Assessment (SOFA) and quick SOFA (qSOFA) scores upon hospital admission. 13 14 15 Additionally, the outcome of each patient regarding in-hospital mortality was recorded. Physiological parameters such as systemic blood pressure, heart rate, respiratory rate, body temperature, use of sedatives, intravenous vasopressors, and anticoagulants were recorded concomitantly to the TCD assessment. 16

**Table 1:** Comorbidities of the study sample

Comorbidity	n/N (%)
Systemic arterial hypertension	17/31 (54.8%)
Diabetes mellitus	14/31 (45.2%)
Chronic kidney disease	9/31 (29.0%)
Cardiopathies	7/31 (22.6%)
Dyslipidemia	7/31 (22.6%)
Smoking	7/31 (22.6%)
Immunosuppression	6/31 (19.4%)
Obesity	5/31 (16.1%)
Neoplasia	4/31 (12.9%)
Asthma	2/31 (6.5%)
Rheumatologic diseases	2/31 (6.5%)
Alcoholism	2/31 (6.5%)
Hematologic diseases	1/31 (3.2%)
Chronic obstructive pulmonary disease	1/31 (3.2%)
Liver disease	1/31 (3.2%)

Laboratory data collected within 48 hours before or after the TCD assessment were included. If not performed within this timeframe, the results were considered unavailable. Variables known to be associated with increased severity/inflammation in COVID-19 patients, as described in the global literature, 16 17 18 were recorded: hemoglobin (Hb), hematocrit (Htc), total leukocytes, total lymphocytes, platelet count, aspartate aminotransferase (AST), alanine aminotransferase (ALT), direct bilirubin (DB), international normalized ratio (INR), activated partial thromboplastin time (aPTT), C-reactive protein (CRP), ferritin, D-dimer, creatine phosphokinase (CPK), and lactate dehydrogenase (LDH).

Arterial blood gas parameters collected on the day of the TCD assessment included: arterial lactate, partial pressure of oxygen (PaO2), partial pressure of carbon dioxide (PaCO2), oxygen saturation (SatO2), arterial oxygen content (CaO2), fraction of inspired oxygen (FiO2), and the PaO2/FiO2 ratio. The CaO2 was calculated using the formula: CaO2 (mL/100 mL) = Hb (mmol/L) × (1.34 mL O2/g Hb) × [(1.61 g Hb/100 mL)/(mmol Hb/L)] × saturation (%/100). 19

### Outcomes

The primary aim of the current study was to assess the frequency of spontaneous cerebral microembolism 8 9 through non-invasive monitoring using TCD in patients with confirmed COVID-19, and to compare the incidence of these events among the 3 study groups.

Additionally, we aimed to evaluate potential differences in TCD parameters, including CBFV in the MCAs, pulsatility, resistance indices of the MCAs, and eCPP. 20 21 We presumed the niICP, 22 and correlated these findings with laboratory results (inflammatory and severity markers) and organ dysfunction mortality predictor scores such as the SOFA and qSOFA. 13 14 15

### Transcranial Doppler

The TCD was performed by a single experienced examiner and reviewed by a certified neurosonologist in all patients using a 2-MHz pulsed-wave transducer (Doppler-Box, Compumedics Germany GmbH). All exams were conducted with the patient in the supine position and the examiner positioned behind the patient's head. Routine TCD was performed using the transtemporal, suboccipital, transorbital, submandibular, and retromastoid windows, followed by continuous bilateral monitoring (for 1 hour) using a TCD helmet, with transducers fixed bilaterally on the transtemporal windows targeting the MCAs. 12 23 The exam was conducted within 72 hours of admission between the first and seventh days of hospitalization.

The TCD variables of interest were: 20 peak systolic, mean, and end-diastolic blood flow velocities in the MCAs (averaged for the right and left MCAs), pulsatility and resistance indices of the MCAs, eCPP, and presumed niICP. Microembolic signals were considered present if they met the following criteria: high-intensity unidirectional signal; short duration (< 300 ms); non-repetitive signal pattern; detection in less than 2 cardiac cycles; and exclusion of artifacts and equipment noise.

An intracranial pressure > 20 mmHg was considered abnormal and indicative of intracranial hypertension. 21 22 23 The eCPP was calculated by multiplying the mean arterial pressure by the ratio of end-diastolic velocity to mean MCA blood flow velocity, adding 14 mmHg. The niICP was calculated as the difference between the eCPP and the patient's mean arterial pressure at the time of the TCD. 20

### Statistical analysis

The qualitative variables were expressed through absolute and relative frequencies and the total number of valid observations. The quantitative variables were expressed through median, first and third quartile, minimum, and maximum values, as well as the number of observations. The distribution of quantitative variables was assessed using boxplots, histograms, and Shapiro-Wilk normality tests for the total sample, revealing that most variables did not follow a normal distribution. 24 To compare TCD data among the study groups, data were expressed through median, first and third quartile, minimum, and maximum values, as well as the number of observations. Nonparametric Kruskal-Wallis tests were applied, assuming the null hypothesis of group equality.

Because the data were non-normally distributed, the relationships among quantitative TCD variables (end-diastolic velocity, mean velocity, peak velocity, pulsatility, and resistance indices) were assessed using Spearman's correlation coefficients (Rho [ρ]). Correlation analyses were performed for the entire cohort (n = 31) to evaluate the relationship between systemic variables and cerebral hemodynamic parameters across all critically-ill patients, regardless of the COVID-19 status, given the exploratory nature of the study and the limited sample size. 24

All analyses were performed using the R (R Foundation for Statistical Computing, Vienna, Austria) software, version 4.0.5.

### Ethical considerations

All patients or their legal representatives provided written informed consent. The study was approved by the Ethics Committee of UNIFESP under protocol 31589920.7.1001.5505, and it was registered for Clinical Trials on April 27, 2021 ( https://clinicaltrials.gov/ct2/show/NCT04861402 ). The study results were unavailable to the treating physicians during the study period and did not influence clinical or therapeutic decisions.

## RESULTS

**Figure 1:**
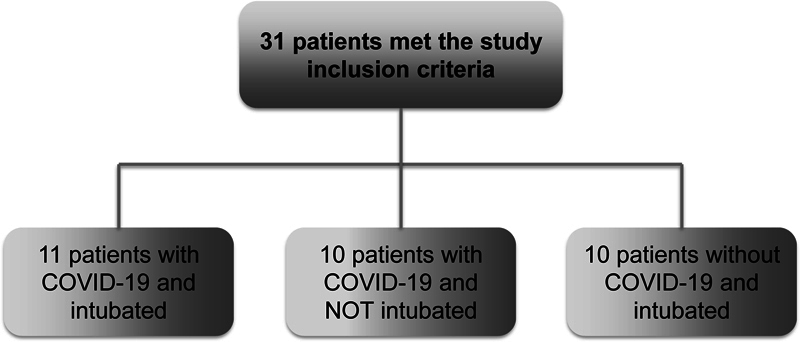
Study design flowchart.

Between July 2020 and August 2021, we evaluated 31 patients: 11 intubated COVID-19 patients, 10 non-intubated COVID-19 patients, and 10 intubated non-COVID-19 patients ( Figure 1 ). The participants' ages ranged from 29 to 84 (median: 62; interquartile range [IQR]: 50–70) years, and most subjects were male (64.5%).

In toal, 17 out of the 31 patients (54.8%) died during hospitalization, and 2 patients (6.45%) had bilateral absence of detectable ultrasound signal through the transtemporal window and were excluded from MCA velocity analyses. No patients presented with unilateral absence of acoustic window. No MESs were detected in any patient. The systemic physiological and laboratory variables of the study sample are presented in **Supplementary Material Table S1** (available at https://www.arquivosdeneuropsiquiatria.org/wp-content/uploads/2026/03/ANP-2025.0202-Supplementary-Material.docx ).

There were no significant differences among the three groups regarding the TCD parameters analyzed ( Table 2 ). Overall, 5 out of the 31 patients (16.12%) presented elevated presumed niICP (> 20 mmHg); of these, 2 were from the intubated COVID-19 group, both of whom died during hospitalization, while 3 were from the non-intubated COVID-19 group. These patients did not experience any neurological complications during their hospital stay, although, after discharge symptom data were unavailable.

**Table 2:** Comparison of TCD data from the groups of interest

Variables		Group 1 (positive COVID-19 and intubated)	Group 2 (positive COVID-19 and non-intubated)	Group 3 (negative COVID-19 and intubated for other disease)	p-value
Peak systolic cerebral blood flow velocity of the MCAs (cm/s)	Median (1st–3rd quartiles); min-max	104.5 (81.5–114.5); 60.0–242,5	87.3 (81.5–113.0); 64.5–136.5	119.0 (95.5–132.5); 77.0–154.0	0.215
Mean cerebral blood flow velocity of the MCAs (cm/s)	Median (1st–3rd quartiles); min-max	55.0 (48.0–75.0); 43.0–162.0	51.0 (46.0–57.0); 41.0–76.0	66.0 (59.0–71.0); 41.5–102.0	0.316
Pulsatility index of the MCAs	Median (1st–3rd quartiles); min-max	0.9 (0.8–1.3); 0.7–1.9	1.2 (1.0–1.3); 0.8–1.8	1.2 (1.0–1.3); 0.9–1.4	0.420
Resistance index of the MCAs	Median (1st–3rd quartiles); min-max	0.5 (0.5–0.8); 0.5–0.8	0.7 (0.6–0.7); 0.5–0.8	0.7 (0.6–0.7); 0.6–0.7	0.156
End-diastolic velocity of the MCAs (cm/s)	Median (1st–3rd quartiles); min-max	40.5 (34.7–60.6); 21.5–109.6	34.4 (30.3–44.4); 29.9–51.0	48.5 (39.7–53.5); 30.5–72.9	0.136
Estimated cerebral perfusion pressure (mmHg)	Median (1st–3rd quartiles); min-max	74.4 (70.3–76.2); 60.2–82.0	82.3 (68.4–94.0); 63.6–100.3	78.8 (73.6–79.3); 58.5–104.0	0.343
(Presumed non-invasive intracranial pressure (mmHg)	Median (1st–3rd quartiles); min-max	7.6 (3.4–14.5); 1.5–44.7	13.5 (5.0–23.6); 4.7–32.9	11.9 (7.5–12.9); 3.2–16.1	0.497

Abbreviations: COVID-19, coronavirus disease 2019; max, maximum; MCA, middle cerebral artery; min, minimum; TCD, transcranial Doppler.

We observed a positive correlation between CBFVs in the MCAs and respiratory rate (Spearman ρ = 0.426; p = 0.021). Additionally, the CBFVs in the MCAs were negatively correlated with ferritin levels (in ng/mL) (Spearman ρ = -0.943; *p* = 0.005) ( Table 3 ). However, ferritin data were only available for six participants. We also observed a positive correlation between the MCAs' resistance index and D-dimer levels (Spearman ρ = 0.390; *p* = 0.049) ( Table 4 ).

**Table 3:** Correlations regarding the mean cerebral blood flow velocity of the MCAs and laboratory test results, vital signs, and organ dysfunction scores

Variables	Spearman correlation with mean cerebral blood flow velocity	*p* -value
Hemoglobin (g/dL)	Spearman ρ = -0.292 (n = 29)	0.124
Hematocrit (%)	Spearman ρ = -0.280 (n = 29)	0.142
Leukocytes (/µL)	Spearman ρ = -0.145 (n = 29)	0.453
Lymphocytes (/µL)	Spearman ρ = -0.027 (n = 29)	0.891
Platelets (/µL)	Spearman ρ = -0.025 (n = 29)	0.900
Urea (mg/dL)	Spearman ρ = -0.042 (n = 29)	0.827
Aspartate aminotransferase (u/L)	Spearman ρ = 0.078 (n = 24)	0.717
Alanine aminotransferase (u/L)	Spearman ρ = -0.182 (n = 25)	0.384
Direct bilirubin (mg/dL)	Spearman ρ = -0.013 (n = 27)	0.949
International normalized ratio	Spearman ρ = -0.205 (n = 24)	0.336
Activated partial thromboplastin time (seconds)	Spearman ρ = -0.233 (n = 24)	0.273
Lactate (mg/dL)	Spearman ρ = 0.024 (n = 27)	0.904
Partial pressure of carbon dioxide (mmHg)	Spearman ρ = 0.065 (n = 29)	0.736
Partial pressure of oxygen (mmHg)	Spearman ρ = 0.356 (n = 29)	0.058
Oxygen saturation (%)	Spearman ρ = 0.314 (n = 29)	0.097
Ferritin (ng/mL)	Spearman ρ = -0.943 (n = 6)	**0.005**
C-reactive protein (mg/L)	Spearman ρ = 0.383 (n = 24)	0.064
Lactate dehydrogenase (u/L)	Spearman ρ = 0.342 (n = 16)	0.195
Troponin-T (pg/mlL	Spearman ρ = 0.236 (n = 24)	0.267
D-dimer (µg/mL)	Spearman ρ = 0.074 (n = 26)	0.719
Creatine phosphokinase (u/L)	Spearman ρ = 0.233 (n = 23)	0.285
Arterial oxygen content (ml/100mL)	Spearman ρ = -0.234 (n = 29)	0.222
Fraction of inspired oxygen	Spearman ρ = -0.071 (n = 29)	0.716
Partial pressure of oxygen/fFraction of inspired oxygen ratio	Spearman ρ = 0.188 (n = 29)	0.329
SOFA score (ICU admission)	Spearman ρ = -0.057 (n = 28)	0.775
QSOFA score (ICU admission)	Spearman ρ = -0.130 (n = 29)	0.502
Systolic blood pressure (mmHg)	Spearman ρ = -0.069 (n = 29)	0.721
Diastolic blood pressure (mmHg)	Spearman ρ = -0.115 (n = 29)	0.554
Mean arterial pressure (mmHg)	Spearman ρ = -0.106 (n = 29)	0.586
Heart rate (bpm)	Spearman ρ = -0.040 (n = 29)	0.836
Respiratory rate (rpm)	Spearman ρ = 0.426 (n = 29)	**0.021**

Abbreviations: MCA, middle cerebral artery; qSOFA, quick Sequential Organ Failure Assessment; SOFA, Sequential Organ Failure Assessment.

Note: Values of *p* in bold indicate statistical significance.

**Table 4:** Correlations regarding the the Resistance Index of the MCAs and laboratory test results, vital signs, and organ dysfunction scores

Variables	Spearman correlation with the Resistance Index	*p* -value
Hemoglobin (g/dL)	Spearman ρ = -0.291 (n = 29)	0.126
Hematocrit (%)	Spearman ρ = -0.320 (n = 29)	0.091
Leukocytes (/µL)	Spearman ρ = -0.074 (n = 29)	0.704
Lymphocytes (/µL)	Spearman ρ = -0.029 (n = 29)	0.882
Platelets (/µL)	Spearman ρ = -0.132 (n = 29)	0.494
Urea (mg/dL)	Spearman ρ = 0.052 (n = 29)	0.788
Aspartate aminotransferase (u/L)	Spearman ρ = 0.174 (n = 24)	0.415
Alanine aminotransferase (u/L)	Spearman ρ = 0.283 (n = 25)	0.170
Direct bilirubin (mg/dL)	Spearman ρ = 0.072 (n = 27)	0.721
International normalized ratio	Spearman ρ = 0.103 (n = 24)	0.630
Activated partial thromboplastin time (seconds)	Spearman ρ = 0.353 (n = 24)	0.090
Lactate (mg/dL)	Spearman ρ = 0.147 (n = 27)	0.464
Partial pressure of carbon dioxide (mmHg)	Spearman ρ = -0.143 (n = 29)	0.459
Partial pressure of oxygen (mmHg)	Spearman ρ = -0.196 (n = 29)	0.309
Oxygen saturation (%)	Spearman ρ = -0.013 (n = 29)	0.946
Ferritin (ng/mL)	Spearman ρ = -0.464 (n = 6)	0.354
C-reactive protein (mg/L)	Spearman ρ = 0.256 (n = 24)	0.227
Lactate dehydrogenase (u/L)	Spearman ρ = 0.153 (n = 16)	0.571
Troponin-T (pg/mlL	Spearman ρ = -0.092 (n = 24)	0.671
D-dimer (µg/mL)	Spearman ρ = 0.390 (n = 26)	**0.049**
Creatine phosphokinase (u/L)	Spearman ρ = 0.242 (n = 23)	0.266
Arterial oxygen content (ml/100mL)	Spearman ρ = -0.305 (n = 29)	0.108
Fraction of inspired oxygen	Spearman ρ = -0.266 (n = 29)	0.163
Partial pressure of oxygen/fFraction of inspired oxygen ratio	Spearman ρ = 0.180 (n = 29)	0.349
SOFA score (ICU admission)	Spearman ρ = -0.085 (n = 28)	0.666
QSOFA score (ICU admission)	Spearman ρ = 0.114 (n = 29)	0.557
Systolic blood pressure (mmHg)	Spearman ρ = 0.132 (n = 29)	0.494
Diastolic blood pressure (mmHg)	Spearman ρ = 0.005 (n = 29)	0.978
Mean arterial pressure (mmHg)	Spearman ρ = 0.085 (n = 29)	0.660
Heart rate (bpm)	Spearman ρ = -0.069 (n = 29)	0.720
Respiratory rate (rpm)	Spearman ρ = -0.209 (n = 29)	0.276

Abbreviations: MCA, middle cerebral artery; qSOFA, quick Sequential Organ Failure Assessment; SOFA, Sequential Organ Failure Assessment.

Note: Values of *p* in bold indicate statistical significance.

Furthermore, the DB, CRP, aPTT, and D-dimer values were negatively correlated with the eCPP (Spearman ρ = -0.494; *p* = 0.009; -0.540; 0.006; -0.495; 0.014; -0.439; and 0.025 respectively) ( Table 5 ).

**Table 5:** Correlation between the estimated cerebral perfusion pressure (e-CPP) and results of laboratory tests, vital signs, and organ dysfunction scores

Variables	Spearman correlation with the e-CPP	*p* -value
Hemoglobin (g/dL)	Spearman ρ = 0.301 (n = 29)	0.113
Hematocrit (%)	Spearman ρ = 0.296 (n = 29)	0.119
Leukocytes (/µL)	Spearman ρ = -0.115 (n = 29)	0.553
Lymphocytes (/µL)	Spearman ρ = -0.063 (n = 29)	0.747
Platelets (/µL)	Spearman ρ = 0.065 (n = 29)	0.737
Urea (mg/dL)	Spearman ρ = -0.300 (n = 29)	0.114
Aspartate aminotransferase (u/L)	Spearman ρ = -0.020 (n = 24)	0.926
Alanine aminotransferase (u/L)	Spearman ρ = 0.045 (n = 25)	0.831
Direct bilirubin (mg/dL)	Spearman ρ = -0.494 (n = 27)	**0.009**
International normalized ratio	Spearman ρ = -0.109 (n = 24)	0.612
Activated partial thromboplastin time (seconds)	Spearman ρ = -0.495 (n = 24)	**0.014**
Lactate (mg/dL)	Spearman ρ = -0.309 (n = 27)	0.117
Partial pressure of carbon dioxide (mmHg)	Spearman ρ = -0.260 (n = 29)	0.173
Partial pressure of oxygen (mmHg)	Spearman ρ = 0.230 (n = 29)	0.230
Oxygen saturation (%)	Spearman ρ = 0.075 (n = 29)	0.699
Ferritin (ng/mL)	Spearman ρ = -0.314 (n = 6)	0.544
C-reactive protein (mg/L)	Spearman ρ = -0.540 (n = 24)	**0.006**
Lactate dehydrogenase (u/L)	Spearman ρ = -0.153 (n = 16)	0.572
Troponin-T (pg/mlL	Spearman ρ = -0.191 (n = 24)	0.372
D-dimer (µg/mL)	Spearman ρ = -0.439 (n = 26)	**0.025**
Creatine phosphokinase (u/L)	Spearman ρ = -0.189 (n = 23)	0.388
Arterial oxygen content (ml/100mL)	Spearman ρ = 0.310 (n = 29)	0.102
Fraction of inspired oxygen	Spearman ρ = -0.227 (n = 29)	0.236
Partial pressure of oxygen/fFraction of inspired oxygen ratio	Spearman ρ = 0.339 (n = 29)	0.072
SOFA score (ICU admission)	Spearman ρ = 0.043 (n = 28)	0.828
QSOFA score (ICU admission)	Spearman ρ = -0.107 (n = 29)	0.581
Systolic blood pressure (mmHg)	Spearman ρ = 0.544 (n = 29)	**0.002**
Diastolic blood pressure (mmHg)	Spearman ρ = 0.504 (n = 29)	**0.005**
Mean arterial pressure (mmHg)	Spearman ρ = 0.619 (n = 29)	**< 0.001**
Heart rate (bpm)	Spearman ρ = -0.080 (n = 29)	0.680
Respiratory rate (rpm)	Spearman ρ = 0.301 (n = 29)	0.113

Abbreviations: qSOFA, quick Sequential Organ Failure Assessment; SOFA, Sequential Organ Failure Assessment.

Note: Values of *p* in bold indicate statistical significance.

## DISCUSSION

The primary finding of the current study was the absence of spontaneous cerebral MESs in the COVID-19 patients, even in those critically ill and requiring invasive MV, which is consistent the with global literature. Importantly, our monitoring protocol employed a 1-hour continuous bilateral TCD evaluation, compared to the 15-minute monitoring duration used by Ziai et al. 19 This extended monitoring period enhances the sensitivity to detect MESs and provides more robust evidence of the absence of spontaneous cerebral microemboli in COVID-19 patients. Only 4 of the 31 patients received continuous intravenous anticoagulation with unfractionated heparin (UFH): 3 from the intubated COVID-19 group and 1 from the intubated non-COVID-19 group. Low-molecular-weight heparin was unavailable at Hospital São Paulo during the pandemic. Therefore, we do not believe that anticoagulation explains the absence of MESs in the current study. In 2000, Lund et al. 25 published a study whose primary objective was to determine the prevalence and frequency of spontaneous cerebral MESs in an unselected sample of patients with acute ischemic stroke (TCD was performed within 72 hours of stroke onset). Microemboli were present in 22 patients (26.5%) of the 83 evaluated with TCD in the study, 25 even though 90% of them were receiving antiplatelet and/or anticoagulant therapy, indicating that the frequency of MESs can be high in acute cerebrovascular conditions. 26

In a study published during the first year of the pandemic by Reynolds et al., 27 microbubbles in the cerebral circulation were detected after agitated saline injection, primarily in most hypoxemic patients, but they did not assess spontaneous microemboli. They performed TCD in 18 intubated patients with severe COVID-19 pneumonia. They found that 83% of the patients had detectable microbubbles, and the number of microbubbles was inversely proportional to the PaO2/FiO2 ratio and pulmonary compliance, suggesting that pulmonary microvascular dilation may be a significant cause of hypoxemia in COVID-19 patients.

In the current series, an unexpected finding was the absence of correlation between hematometric levels (hemoglobin and hematocrit) and CBFVs, despite the fact that this relationship has been well-established in previous long-standing studies. It is known that the lower the hematocrit, the lower the blood viscosity, and the higher the CBFVs. While Ziai et al. 19 reported an inverse correlation between hematocrit and CBFVs in COVID-19 patients, the lack of correlation in our cohort may reflect the relatively-narrow hematocrit range observed, or other confounding factors related to critical illness that warrant further investigation. Another unexpected finding was the positive correlation between MCA blood flow velocities and respiratory rate. Generally, higher respiratory rates are associated with lower CBFVs, as hyperventilation leads to cerebral vasoconstriction. Additionally, the absence of correlation between CBFVs and the PaO2/PaCO2 ratio is noteworthy, given the well-established relationship between carbon dioxide and cerebral blood flow. This lack of expected physiological correlation may reflect impairment of cerebral blood flow regulation mechanisms in critically-ill patients, potentially attributable to systemic metabolic derangements or undetected cerebral ischemic processes. Unfortunately, serial blood gas measurements during the monitoring period were not available, precluding analysis of the temporal variability of O2 and CO2 in relation to CBFV, which could have provided additional pathophysiological insights.

The DB, CRP, aPTT, and D-dimer values were negatively correlated with the eCPP. While these findings suggest that patients with more pronounced inflammatory and coagulation abnormalities may have lower cerebral perfusion, the absence of correlation between the eCPP and the clinical severity indices (SOFA, qSOFA) warrants cautious interpretation. Given the small sample size and the exploratory nature of these analyses, these correlations should be considered hypothesis-generating, and they require validation in larger cohorts. Coagulation abnormalities are a well-recognized complication of COVID-19, 28 including elevated D-dimer and fibrinogen levels. Typical laboratory findings in hospitalized COVID-19 patients include lymphopenia, thrombocytopenia, elevated aminotransferases, LDH, CPK, troponin, D-dimer, and inflammatory markers (such as interleukin-6 [IL-6], interleukin-1 [IL-1], erythrocyte sedimentation rate [ESR], ferritin, and CRP), 29 as well as coagulation test abnormalities, findings that are also related to greater severity and worse prognosis in these patients. 30

### Limitations

The present study has several limitations that warrant consideration. First, the small sample size (n = 31) limits statistical power and the generalizability of our findings, and the study should be considered exploratory. Second, the current was was a single-center study conducted during the initial waves of the COVID-19 pandemic, and findings may not apply to different viral variants or evolving treatment protocols. Third, the eCPP formula used in the present study was originally validated in patients with traumatic brain injury (TBI), and its accuracy in non-TBI critically-ill populations requires further validation. Fourth, we did not collect serial arterial blood gas measurements during the 1-hour TCD monitoring, precluding analysis of temporal relationships involving gas exchange parameters and cerebral hemodynamics. Fifth, the inclusion of non-COVID-19 patients with heterogeneous diagnoses in the correlation analyses may have introduced confounding. Sixth, the presumed niICP analysis yielded no significant findings, which should be interpreted with caution, given the lack of validation of this parameter in this population. Finally, long-term neurological outcomes were not assessed, limiting our ability to correlate TCD findings with clinical sequelae.

In conclusion, the current exploratory study supports the existing literature, indicating that, despite the increased incidence of thromboembolic events in COVID-19 patients, spontaneous cerebral microemboli are not frequent during prolonged TCD monitoring, regardless of the need for or type of ventilatory support. Therefore, other pathophysiological mechanisms that explain the neurovascular manifestations of COVID-19 should be considered. Sicker patients with higher DB, CRP, aPTT, and D-dimer levels had lower eCPP, although these correlations require validation. Future multicenter studies with larger sample sizes and systematic TCD protocols in neurointensive care settings are warranted to elucidate further cerebral hemodynamic alterations in COVID-19 and other severe infectious diseases.
